# IRE1α siRNA relieves endoplasmic reticulum stress-induced apoptosis and alleviates diabetic peripheral neuropathy *in vivo* and *in vitro*

**DOI:** 10.1038/s41598-018-20950-9

**Published:** 2018-02-07

**Authors:** Weijie Yao, Xinwei Yang, Jiayue Zhu, Biane Gao, Haotian Shi, Liping Xu

**Affiliations:** School of Traditional Chinese Medicine, Capital Medical University, Beijing, China

## Abstract

Diabetic peripheral neuropathy (DPN) is mainly characterized by demyelination resulted from the apoptosis of the Schwann cell (SCs). Although the exact mechanisms underlying DPN remain unclear, endoplasmic reticulum (ER) stress is strongly implicated in the apoptosis. Under ER stress, activated inositol-requiring kinase 1α (IRE1α) unregulated CHOP, phosphorylated JNK and Caspase-12 to aggravate apoptosis-mediated damage of DPN. Therefore, we tested the hypothesis that inhibition of IRE1α could reduce the ER stress-related apoptosis to relieve DPN. Here, we show that IRE1α siRNA improved the neurological morphology and function of DPN rats and rescued ER stress-related apoptosis in the sciatic nerve. Additionally, RSC96 cells transfected with IRE1α siRNA were used as *in vitro* model of DPN. It was found that IRE1α siRNA also decreased high glucose-induced apoptosis and inhibited ER stress-related apoptosis in the cells. Altogether, our results suggest that IRE1α should be considered a potential therapeutic agent for DPN.

## Introduction

Diabetic peripheral neuropathy (DPN), one of the diabetes complications, is a neurodegenerative disease with no treatment options available^1^. Currently, Schwannopathy is considered to be one of the integral factors in the pathogenesis of DPN^2^. Chronic hyperglycemia induces endoplasmic reticulum (ER) stress, which is the key factor leading to the apoptosis of the Schwann cell (SCs), and contributing to DPN^3^.

ER is the major organelle for the translocation, folding, and post-translational modifications of proteins. Perturbations in ER function, a process named ER stress, trigger unfolded protein response (UPR), a complementary adaptive machinery, organized by three ER transmembrane receptor proteins. Inositol-requiring kinase 1α (IRE1α), double-stranded RNA-activated protein kinase (PKR)-like endoplasmic reticulum kinase (PERK) and activating transcription factor 6 (ATF6) are against ER stress to restore protein homeostasis^4^. If the stress is too severe and excesses the capacity of UPR defense mechanisms, cells switch to apoptotic cell mechanisms^5^.

IRE1α determines cell fate based on ER stress severity. Under unrelieved ER stress, IRE1α degrades many ER-localized mRNAs to induce apoptosis. Hyperglycemia and hyperlipidemia in diabetes patients hyperactivate IRE1α and potentiate ER stress-induced apoptosis^6^. Our previous study has shown that ER stress-induced reactive oxygen species (ROS) is a unified pathological process of DPN *in vivo* and *in vitro*, and ROS feedback induces the IRE1α, thereby aggravating DPN^7–9^. The high sensitivity of IRE1α to hyperglycemia implies that it plays important role in DPN-induced apoptosis^10,11^. However, how the IRE1α pathway is mediated in DPN is not fully clarified. In addition, the inhibitor of IRE1α or IRE1α gene knockdown has been used to intervene diabetes or other metabolic disorders^6,12^. Therefore, this research will focus on the IRE1α pathway in DPN rats and the Schwann cells to investigate whether IRE1α siRNA could relieve DPN as well as the molecular mechanisms underlying ER stress-related apoptosis. IRE1α is expected to be a potentially valuable target for improving DPN.

In this work we evaluated IRE1α inhibition to decrease the ER stress-induced apoptosis, using experimental models of DPN. Here, we demonstrate that inhibition of IRE1α reduces the CHOP, Caspase-12 and phosphorylated JNK related-apoptosis and decreases the demyelination of DPN, potentially leading to new therapeutic approaches.

## Results

### Intrathecal injection siRNA decreased IRE1α in the sciatic nerve of DPN rats

**Figure 1:**
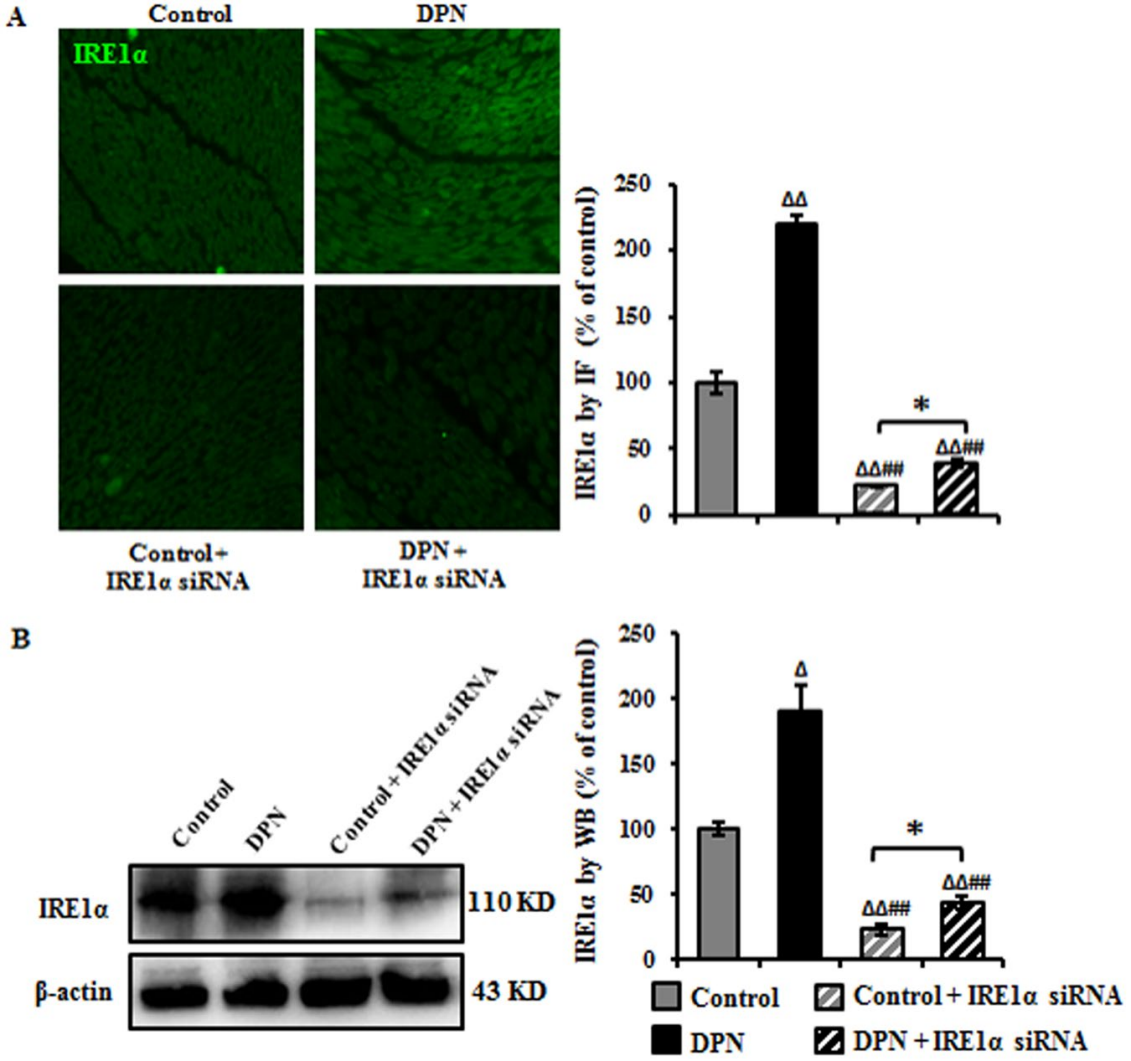
Down-regulation of IRE1α in the sciatic nerve after intrathecal injection of siRNA. (**A**) IRE1α was effectively reduced as measured by immunofluorescence. IRE1α was detected with an antibody against IRE1α, followed by a fluorescent-labeled secondary antibody (green). Data are expressed as mean ± SEM as the percentage of the respective controls and analyzed using one-way ANOVA with LSD analysis or unpaired Student’s t-test. (**B**) Representative Western blots using tissue extracts from the sciatic nerve and probed with antibodies against IRE1α. β-actin was used as loading control. Data are expressed as mean ± SEM indicated as the percentage of the respective controls and analyzed using Tamhane’s T2 analysis or unpaired Student’s t-test. ^Δ^*P* < 0.05, ^ΔΔ^*P* < 0.01, compared to control; ^##^*P* < 0.01, compared to DPN; *P < 0.05 (N = 6 per group).

Our previous study has demonstrated that IRE1α is overexpressed in the sciatic nerve of high-carbohydrate/high-fat diet and low-dose STZ-induced DPN rats and this result was further verified in this study (Fig. 1A), where overexpressed IRE1α induced the prolonged ER stress, thus leading to demyelination and nerve dysfunction^13^. Intrathecally injected IRE1α siRNA was used to further investigate the IRE1α pathway in DPN. Because this method has not been fully reported in DPN rats, immunofluorescence (IF) staining and Western blotting (WB) were used to examine IRE1α expression in the sciatic nerve. 3 days after injection, IRE1α was reduced to 22% (IF staining) and 24% (WB), respectively, as compared to IRE1α siRNA control-transfected group and to 39% (IF staining) and 44% (WB) in DPN IRE1α siRNA transfected group compared to DPN group (Fig. 1). These results suggest that intrathecal injection of IRE1α siRNA downregulates the expression of IRE1α in the sciatic nerve.

### IRE1α siRNA improved neurological morphology and function of DPN rats

**Figure 2:**
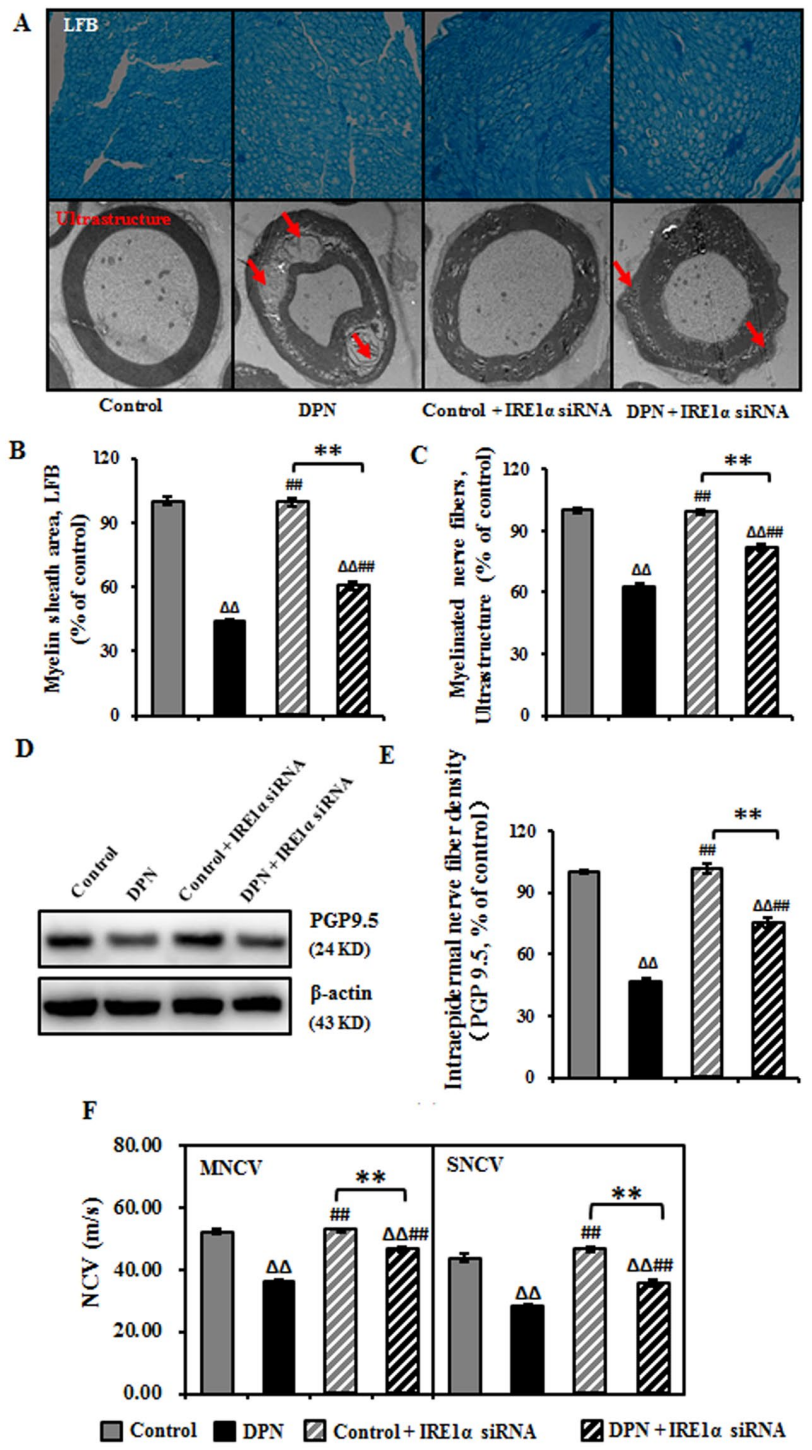
Improved neurological morphology and function of sciatic nerve in DPN rats after the intrathecal injection of IRE1α siRNA. (**A**) Transfection of IRE1α siRNA increased the myelin sheath area and myelinated nerve fibers in DPN rats. Representative pictures of Luxol fast blue (LFB) staining (magnification 40×) of myelin sheath and ultrastructure (magnification 6000×) of myelinated nerve fibers. (**B**) Myelin sheath area expressed as mean ± SEM of the percentage of the respective controls and analyzed using one-way ANOVA with LSD analysis or unpaired Student’s t-test. (**C**) The number of myelinated nerve fibers expressed as mean ± SEM of the percentage of the respective controls and analyzed using one-way ANOVA with LSD analysis or unpaired Student’s t-test. (**D**) Representative Western blots using tissue extracts from the sciatic nerve and probed with antibodies against PGP9.5. β-actin was probed as loading control. (**E**) The intensity of PGP9.5 expressed as mean ± SEM of the percentage of the respective controls and analyzed using one-way ANOVA with LSD analysis or unpaired Student’s t-test. (**F**) NCV reported as mean ± SEM and analyzed using one-way ANOVA with LSD analysis or unpaired Student’s t-test. ^ΔΔ^*P* < 0.01, compared to control; ^##^*P* < 0.01, compared to DPN; **P < 0.01 (N = 6 per group).

To verify if IRE1α siRNA could alleviate DPN, we investigated the morphological and function of the peripheral nerves with a multimodal approach. At the pathological level, as shown in Fig. 2A, the myelin sheath and nerve fibers were reduced to 63% and 44%, respectively, in DPN rats. In the DPN rats transfected with IRE1α siRNA the myelin sheath and nerve fibers increased to 82% and 60%, respectively (Fig. 2B,C). In the DPN rats, there was a significant decrease of the number of cutaneous unmyelinated fibers, as evaluated by intraepidermal nerve fiber density (IENFD) assessment (reduced to 47%), but after IRE1α siRNA transfection, the IENFD increased 0.75-fold (Fig. 2D,E). The MNCV and SNCV in DPN rats were reduced compared to control rats, while IRE1α siRNA treatment increased the MNCV and SNCV compared to DPN rats (Fig. 2F).

### IRE1α siRNA alleviated ER stress-related apoptosis in the sciatic nerve of DPN rats

ER stress activates the UPR signaling pathways that determine cell fate. Remediable ER stress activates adaptive-UPR outputs that favor cell survival, but chronic ER stress activates terminal-UPR (T-UPR) outputs to trigger apoptosis. T-UPR is sufficient to drive IRE1a autophosphorylation, XBP1 mRNA splicing, induction of apoptosis^6^.

**Figure 3:**
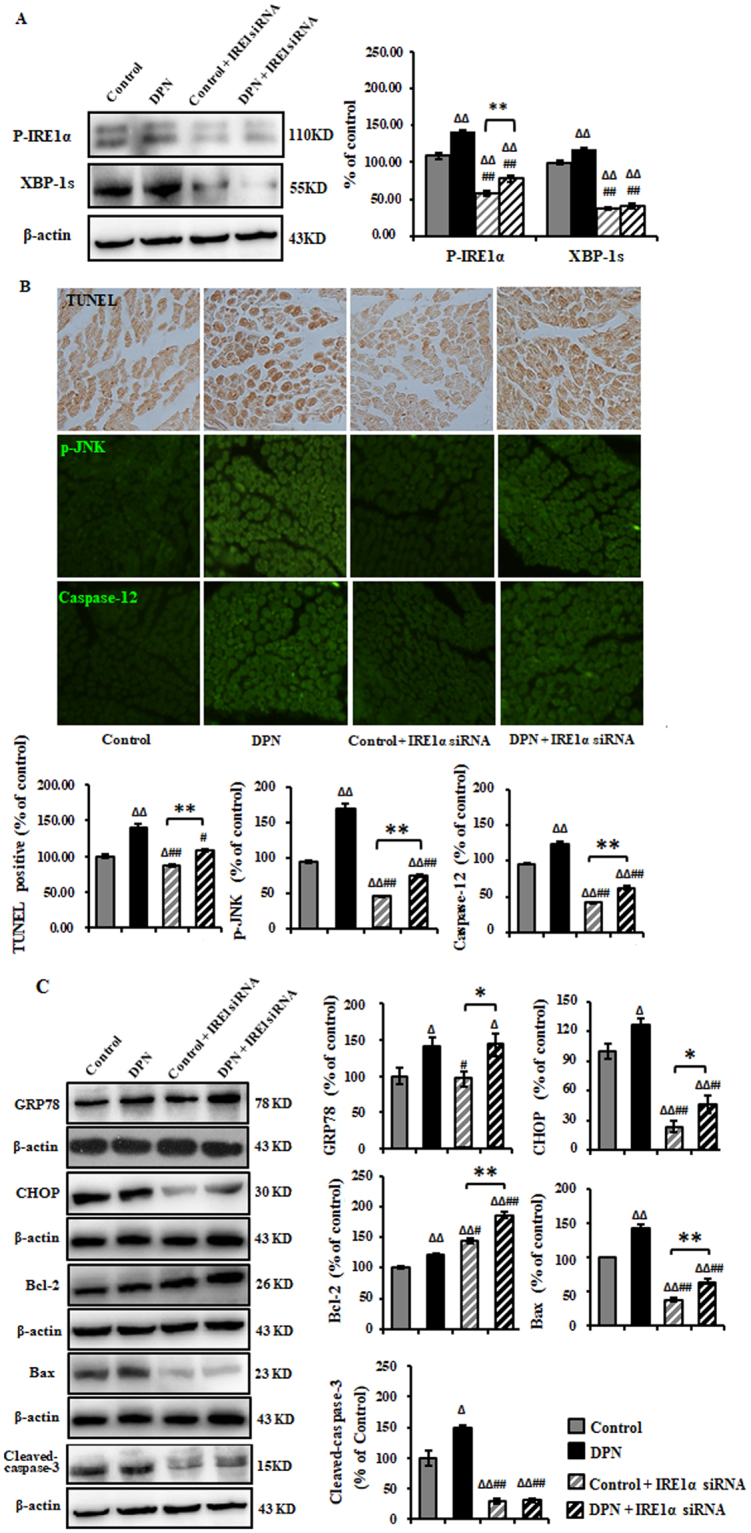
Inhibition of ER stress-related apoptosis in the sciatic nerve of DPN rats by intrathecal injection of IRE1α siRNA. (**A**) Representative Western blots using tissue extracts from the sciatic nerve and probed with antibodies against P-IRE1α and XBP-1s. β-actin was probed as loading control. The intensity is expressed as mean ± SEM of the percentage of the respective controls and analyzed using one-way ANOVA with LSD analysis or unpaired Student’s t-test. (**B**) TUNEL, p-JNK, and Caspae-12 were effectively eliminated as measured by immunohistochemistry method. Data are expressed as mean ± SEM of the percentage of the respective controls and analyzed using one-way ANOVA with Tamhane’s T2 analysis or unpaired Student’s t-test. (**C**) Representative Western blots using tissue extracts from the sciatic nerve and probed with antibodies against GRP78, CHOP, Bcl-2, Bax and Cleaved-Caspase-3. β-actin was probed as loading control. Results of are expressed as mean ± SEM indicated as percentage of the respective controls and analyzed using one-way ANOVA with LSD analysis (GRP78, Cleaved-Caspase-3, CHOP) or Tamhane’s T2 analysis (Bcl-2, Bax) or unpaired Student’s t-test. ^Δ^*P* < 0.05, ^ΔΔ^*P* < 0.01, compared to control; ^#^*P* < 0.05, ^##^*P* < 0.01, compared to DPN; *P < 0.05, **P < 0.01 (N = 6 per group).

ER stress-induced apoptosis has been reported to be a critical event in DPN, and IRE1α autophosphorylation resulted from hyperactivation is necessary and sufficient to trigger the apoptosis^5^. We therefore examined the phosphorylation state of IRE1 α. IRE1α became 134% activation-loop autophosphorylated in DPN rats compared to control rats, where the expression of XBP-1s increased to 117%. While the depletion IRE1α in the DPN rats down-regulated the expression of p-IRE1 α and XBP-1 expression by 51% and 65%, respectively (Fig. 3A). We hypothesized that deactivation of IRE1α/XBP-1 may inhibit apoptosis in DPN. The TUNEL-positive area in the sciatic nerve of DPN rats increased 40% compared to control rats. The expression of Cleaved-Caspase-3 was up-regulated to 150%, while the knock down of IRE1α in the DPN rats reduced the TUNEL-positive area and expression of cleaved-Caspase-3 to 108% and 31%, respectively (Fig. 3B,C).

Apoptosis-related proteins in the IRE1α/XBP-1 pathway was examined including CHOP, p-JNK and Caspase-12^13^. Results showed that the expression of CHOP, p-JNK and Caspase-12 were increased to 127%, 170%, and 238% in the DPN rats compared to control rats, and the depletion of IRE1α in the DPN rats reduced the levels of CHOP, p-JNK and Caspase-12 to 47%, 75%, and 63%. However, IRE1α siRNA didn’t affect the GRP78 expression. CHOP has been shown to down-regulate Bcl-2 while increase the translocation of Bax from cytosol to mitochondria, which consequently triggers apoptosis. In the DPN rats, the expression of Bcl-2 and Bax increased to 122% and 143%, respectively, compared to control rats. The depletion of IRE1α in DPN rats reduced the expression of Bax to 64%, while increased Bcl-2 to 186%, as compared to control (Fig. 3B,C).

### IRE1α siRNA decreased high glucose-induced hyperactivation of IRE1α/XBP-1s in RSC96 cells

**Figure 4:**
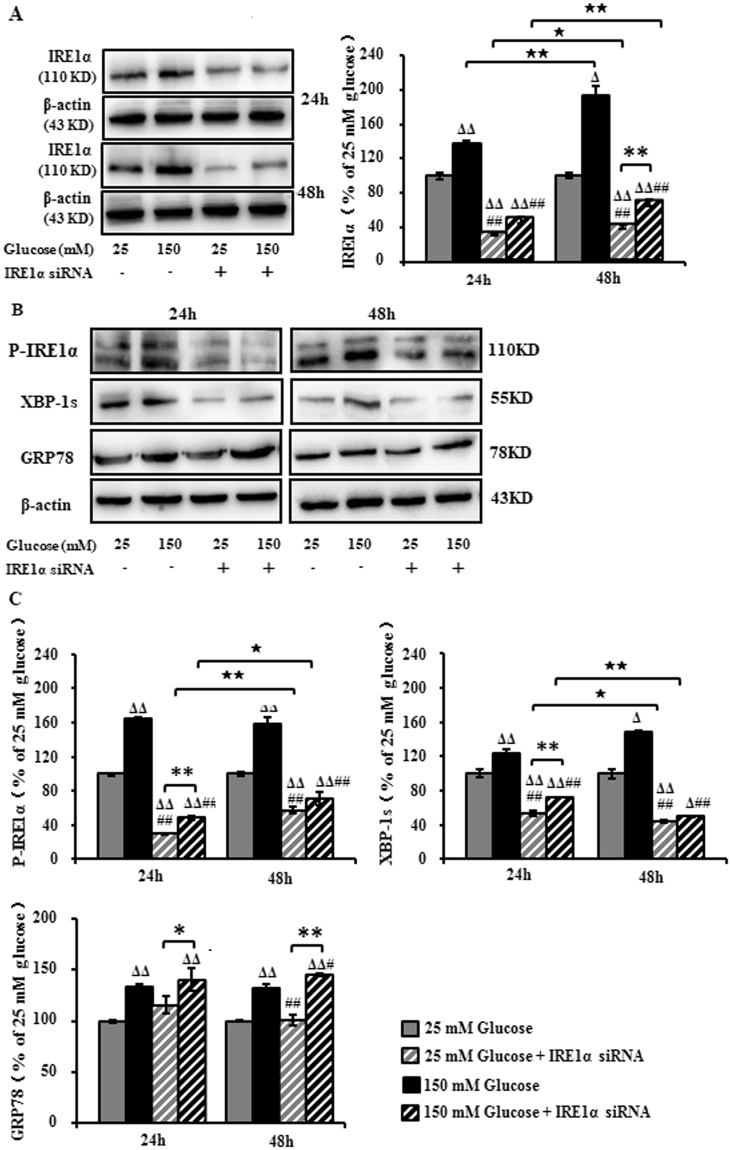
Expression of IRE1α, XBP-1s and GRP78 in RSC96 cells transfected with IRE1α siRNA and exposed to high glucose. (**A**–**C**) Representative Western blots using RSC96 cells and probed with antibodies against IRE1α, P- IRE1α, XBP-1s and GRP78. Results are expressed as mean ± SEM of the percentage of the respective 25 mM glucose group and analyzed using one-way ANOVA analysis. ^Δ^*P* < 0.05, ^ΔΔ^*P* < 0.01, compared to 25 mM glucose; ^#^*P* < 0.05, ^##^*P* < 0.01, compared to 150 mM glucose; ^★^*P* < 0.05, ^★★^*P* < 0.01, Student’s unpaired t-test compared to 24 hours’ time point. *P < 0.05, **P < 0.01 (n = 4 per group).

Our previous study has demonstrated that high glucose in the Schwann cell-rich sciatic nerve plays a key role in the pathogenesis of DPN^7^. To further confirm the effects of IRE1α siRNA on DPN, high glucose-treated Schwann cells (SCs) were used for investigation. For this purpose, RSC96 cells were transfected with IRE1α siRNA followed by exposure to high level of glucose (150 mM) for 24 and 48 hours. IRE1α was persistently up-regulated to 138% and 194% after the cells were incubated with 150 mM glucose for 24 and 48 hours. Transfection of IRE1α siRNA down-regulated the expression to 32% and 48% in 25 mM glucose and 150 mM glucose at 24 h; and to 40% and 69% at 48 h, respectively, suggesting that the expression of IRE1α in RSC96 cells was downregulated (Fig. 4A).

The expression of p-IRE1α and XBP-1s treated with 150 mM glucose was sustainably increased to 165%, 159% (p-IRE1α) and 124%, 149% (XBP-1s), respectively, compared to untreated cells at 24 and 48 h, while the transfection of IRE1α siRNA reduced the expression of p-IRE1α and XBP-1s to 48%, 70% (p-IRE1α) and 71%, 49% (XBP-1s) respectively. On the contrast, the expression of GRP78 was not affected (Fig. 4B,C).

### IRE1α siRNA decreased high glucose-induced apoptosis and Ca^2+^ level in RSC96 cells

**Figure 5:**
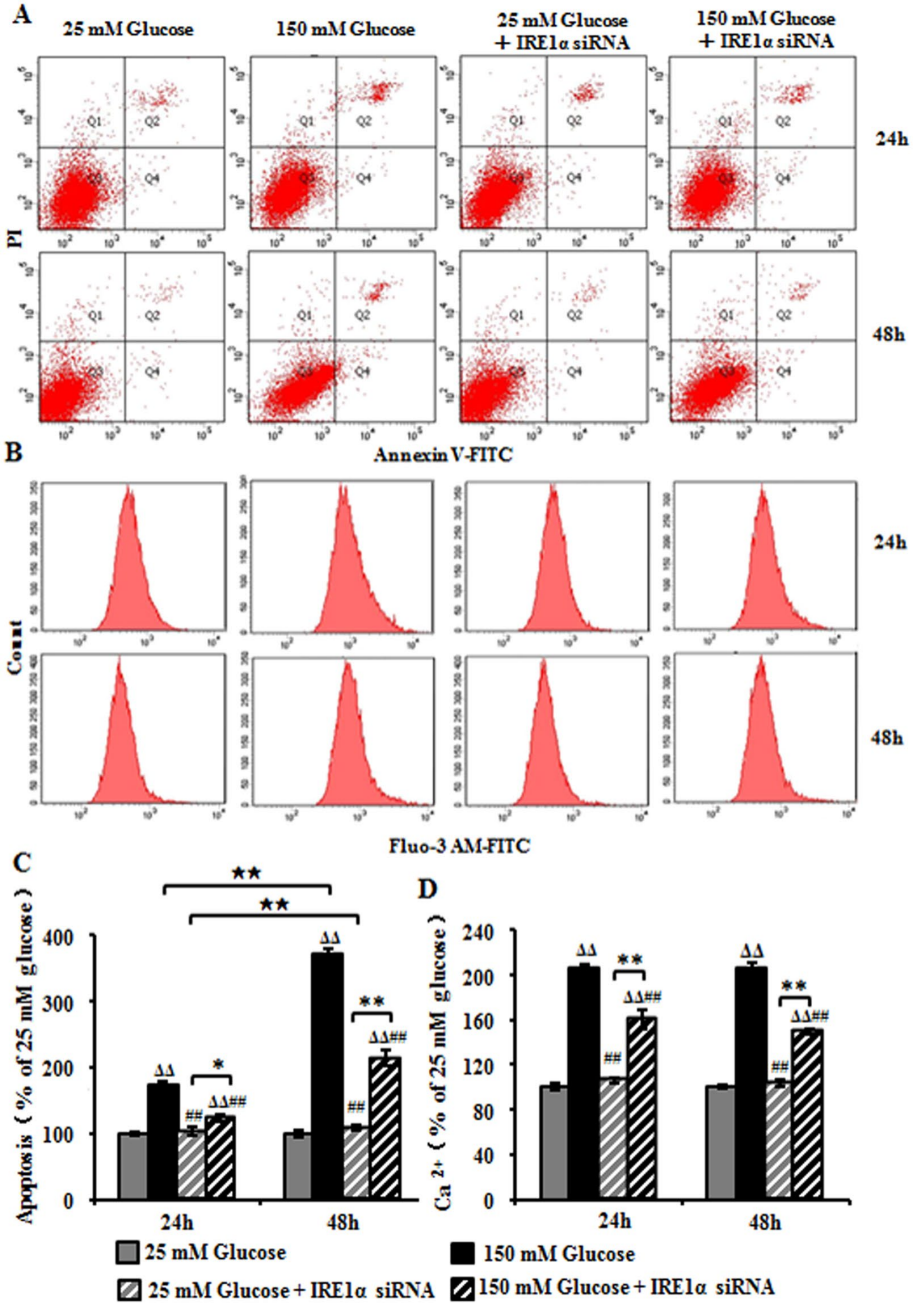
High glucose-induced apoptosis and Ca^2+^ levels in RSC96 cells after IRE1α siRNA transfection. (**A**) FACS apoptosis assay results using FITC-Annexin V and PI staining at 24 h and 48 h. (**B**) Intracellular Ca^2+^ production measured with FACS analysis using Fluo-3 AM dye at 24 h and 48 h. (**C**) Apoptosis levels indicated as the percentage of the respective 25 mM glucose-treated cells. Data are expressed as mean ± SEM and analyzed using one-way ANOVA with LSD analysis. (**D**) Intracellular Ca^2+^ levels indicated as the percentage of the respective 25 mM glucose-treated cells. Data are expressed as mean ± SEM and analyzed using one-way ANOVA with LSD analysis. ^Δ^*P* < 0.05, ^ΔΔ^*P* < 0.01, compared to25 mM glucose; ^##^*P* < 0.01, compared to 150 mM glucose; ^★^*P* < 0.05, ^★★^*P* < 0.01, Student’s unpaired t-test compared to 24 hours’ time point. *P < 0.05, **P < 0.01 (n = 4 per group).

Flow cytometry showed that cells treated with 150 mM glucose had 1.73-fold increase in apoptosis as compared with those treated with 25 mM glucose after 24 hours of exposure, while the pretreatment of IRE1α siRNA reduced the increase to 122%. After 48 hour exposure with 150 mM glucose, the apoptosis increase to 370% as compared with 25 mM glucose, while the pretreatment of IRE1α siRNA decreased the increase to 214% (Fig. 5A,C). We further studied whether IRE1α knock down could affect the production of intracellular Ca^2+^ in RSC96 cells. FACS analysis showed that Ca^2+^ level increased to 206% and 207% after incubation in 150 mM glucose for 24 h and 48 h as compared with 25 mM glucose. However, the pretreatment of IRE1α siRNA redued the Ca^2+^ production to 161% and 150% at 24 h and 48 h compared with 150 mM glucose (Fig. 5B,D).

### IRE1α siRNA inhibited high glucose-induced ER stress-related apoptosis in RSC96 cells

**Figure 6:**
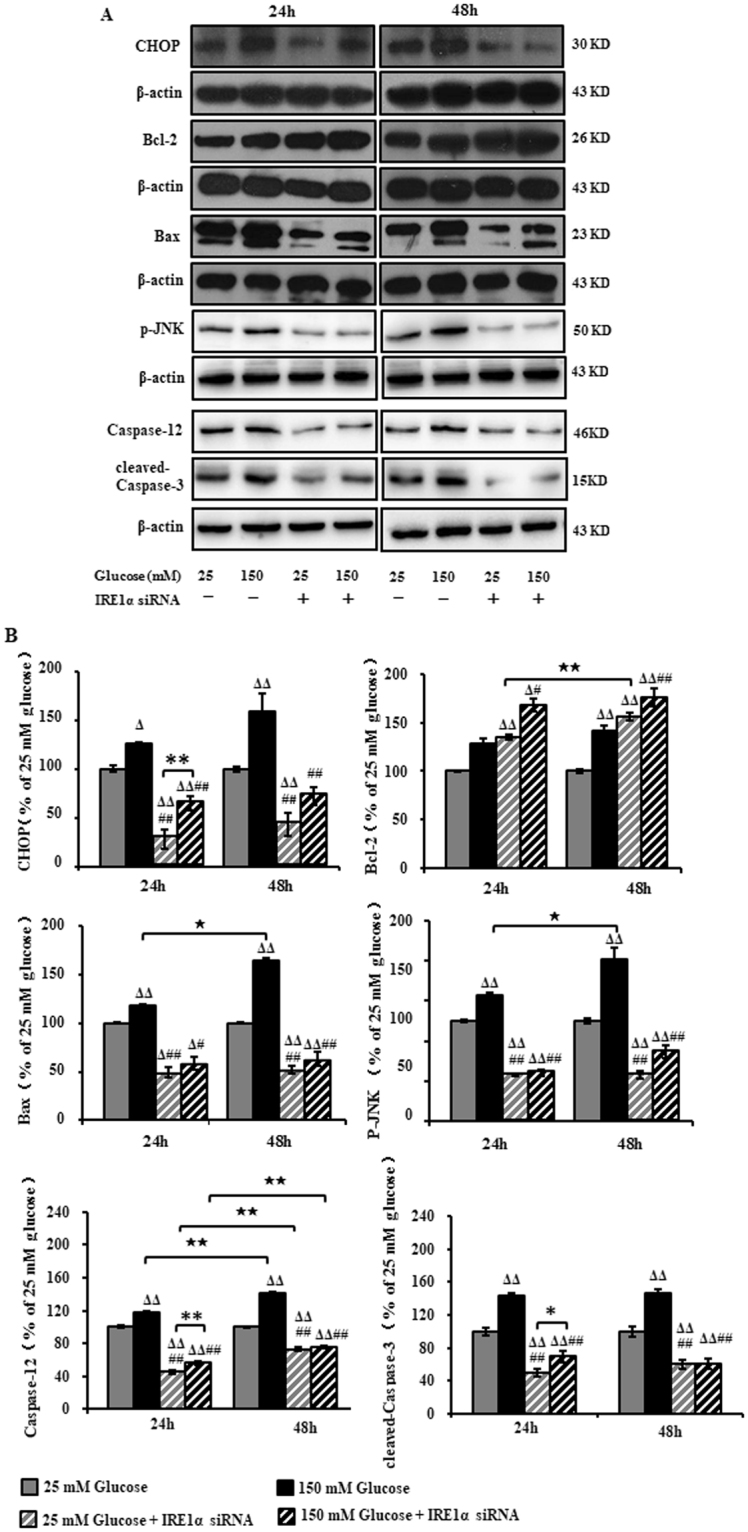
Expression of CHOP, Bcl-2, Bax, p-JNK, Caspase-12 and cleaved-Caspase-3 in RSC96 cells after IRE1α siRNA transfection and exposure to high glucose. (**A**) Representative Western blots using tissue extracts from the sciatic nerve and probed with antibodies against CHOP, Bcl-2, Bax, p-JNK, Caspase-12 and cleaved-Caspase-3. (**B**) The band intensity expressed as mean ± SEM of the percentage of the respective 25 mM glucose-treated cells and analyzed using one-way ANOVA with LSD analysis (CHOP, Bcl-2/48 h, Bax/48 h, p-JNK/24 h, Caspase-12, cleaved-Caspase-3,) or Tamhane’s T2 analysis (p-JNK/48 h, Bcl-2/24 h, Bax/24 h, Caspase-3/24 h,). ^Δ^*P* < 0.05, ^ΔΔ^*P* < 0.01, compared to 25 mM glucose; ^#^*P* < 0.05, ^##^*P* < 0.01, compared to 150 mM glucose; ^★^*P* < 0.05, ^★★^*P* < 0.01, Student’s unpaired t-test compared to 24 hours’ time point. *P < 0.05, **P < 0.01 (n = 4 per group).

To characterize the apoptosis after exposure to high glucose, we measured the expression of CHOP, p-JNK and Caspase-12 and cleaved-Caspase-3. Western analyses showed that after exposure to high glucose for 24 hours and 48 hours, the expression of CHOP was up-regulated to 126% and 159%, respectively, and IRE1α siRNA transfection reduced the expression to 64% and 72%, respectively. The expression of p-JNK was up-regulated to 126% and 162% and IRE1α siRNA transfection reduced the expression to 59% and 51%. The expression of Caspase-12 was up-regulated to 118% and 141% and IRE1α siRNA transfection reduced the expression to 56% and 76%. The expression of cleaved-Caspase-3 was up-regulated to 143% and 146% and IRE1α siRNA transfection reduced the expression to 69% and 61%. We found that Bax was up-regulated as compared with untreated cells, whereas IRE1α siRNA transfection decreased the expression of Bax and further increased the level of Bcl-2 (Fig. 6). Taken together, these observations suggest that IRE1α siRNA specifically inhibits ER stress-induced apoptosis after exposure to high glucose.

## Discussion

Our data show that intrathecal injection of IRE1α siRNA resulted in about 77% and 65% reduction of apoptosis in rats and the SCs, respectively; inhibition of IRE1α improved the demyelination of sciatic nerve, enhanced the MNCV and SNCV, as well as intraepidermal nerve fiber density. In *in vitro* experiment, IRE1α siRNA transfection inhibited apoptosis and Ca^2+^ levels in the SCs. These might be attributed to down-regulation of IRE1-related apoptosis proteins including CHOP and p-JNK in DPN.

Hyperglycemia and dyslipidemia have been identified as activators of ER stress in DPN^14^. Here, high-carbohydrate/high-fat diet and low-dose streptozotocin were used to construct a DPN rat model. Our previous study has demonstrated that in DPN rats the levels of total cholesterol, triglyceride and low-density lipoprotein are increased and the rats become insulin resistant^15^. The characteristic features of DPN include demyelination and axonal atrophy, which lead to NCV deficiency. Schwannopathy is an integral factor in the pathogenesis of DPN^2^. SC dysfunction has direct effects on neuronal function as a result of myelin disruption, demyelination, and changes in NCV. SCs are the glial cells responsible for producing the myelin sheath in the PNS and are highly susceptible to ER stress, because ER is the major organelle for controlling the production of cholesterol and lipid-membrane biosynthesis^16,17^. Therefore, high glucose-treated SCs were used for investigation. Our *in vivo* results reported here and in our previous publication have demonstrated that SC apoptosis results in demyelination, and then reduces the NCV and IENFD.

In the sciatic nerve of DPN rats and SCs exposed to high glucose, GRP78 was up-regulated^7^. GRP78 is triggered and overexpressed when ER stress occurs^14^. Our results showed that inhibition of IRE1α didn’t change the expression of GRP78, suggesting that the inhibition may alter the status of ER stress in DPN and alleviate DPN through reducing the expression of IRE1α-induced apoptosis genes.

Recently, targeting the IRE1α pathway has emerged as a potential approach for treatment of diabetes and its complications^18^. The ER is the main organelle for calcium storage, protein folding and processing, as well as for lipid biosynthesis and metabolism. Metabolic perturbations such as advanced glycation of proteins and lipids, oxidative stress, that are often seen in diabetes and related disorders, lead to ER stress. ER stress activates UPR signaling pathways that determine cell fate.

Remediable ER stress activates adaptive-UPR outputs that favor cell survival. A major advance in understanding the involvement of ER stress in the pathobiology of metabolic disorders has been the discovery of the XBP1, a modulator of glucose and lipid metabolism^12^. Spliced X-box binding protein 1 (sXBP1) translocates to the nucleus to initiate transcription of chaperone proteins and proteins involved in ER-associated protein degradation (ERAD). These adaptive mechanisms of the UPR function to attenuate mild to moderate ER stress to restore normal ER function.

But chronic ER stress activates T-UPR outputs will trigger apoptosis. During times of extreme or chronic stress, for example sustained hyperglycemia, the capacity of the UPR is overwhelmed, induces the T-UPR, and drive IRE1a autophosphorylation and XBP1 mRNA splicing. The IRE1α/XBP-1 pathway is dominant to promote apoptosis^6,11,13^.

Our results show that CHOP was upregulated in the DPN rats and Schwann cells and IRE1α inhibition suppressed the CHOP expression. CHOP plays a critical role in ER stress-induced apoptosis and it is believed to play a central role in ER stress-induced cell death. It is strongly induced via IRE1 signaling^19^.

In addition, IRE1α triggers several other proapoptotic signaling pathways, including ER-resident Caspase-12. Caspase-12 is located on the ER and activated in ER stress-induced apoptosis, but not by membrane- or mitochondrial-targeted apoptotic signals. It has been shown to be important in apoptosis because it is elicited by ER stress in neurodegenerative disease^20,21^. Our present study shows that the inhibition of IRE1α markedly decreased Caspase-12. Previous studies have demonstrated that cleaved-Caspase-3 is involved in ER stress-induced apoptosis and is activated by Caspase-12. Our observations confirmed that cleaved-Caspase-3 was markedly reduced when Caspase-12 was downregulated as a result of IRE1α siRNA transfection.

IRE1α forms a complex with TRAF2 and ASK1, and in turn it recruits and activates JNK^14^. The activation of JNK pathway has been shown to be a common phenomenon in stress-induced apoptosis in response to intracellular stresses and activates Bax^22^. In this research, IRE1α inhibition resulted in reduced JNK activation and then suppressed Bax expression. In addition, CHOP also down regulates Bcl-2 expression and increases the translocation of Bax from the cytosol to mitochondria, which consequently triggers the activation of caspase-3, resulting in apoptosis^23^. Taken together, ER stress-induced apoptosis is relieved by IRE1α inhibition^24^.

**Figure 7:**
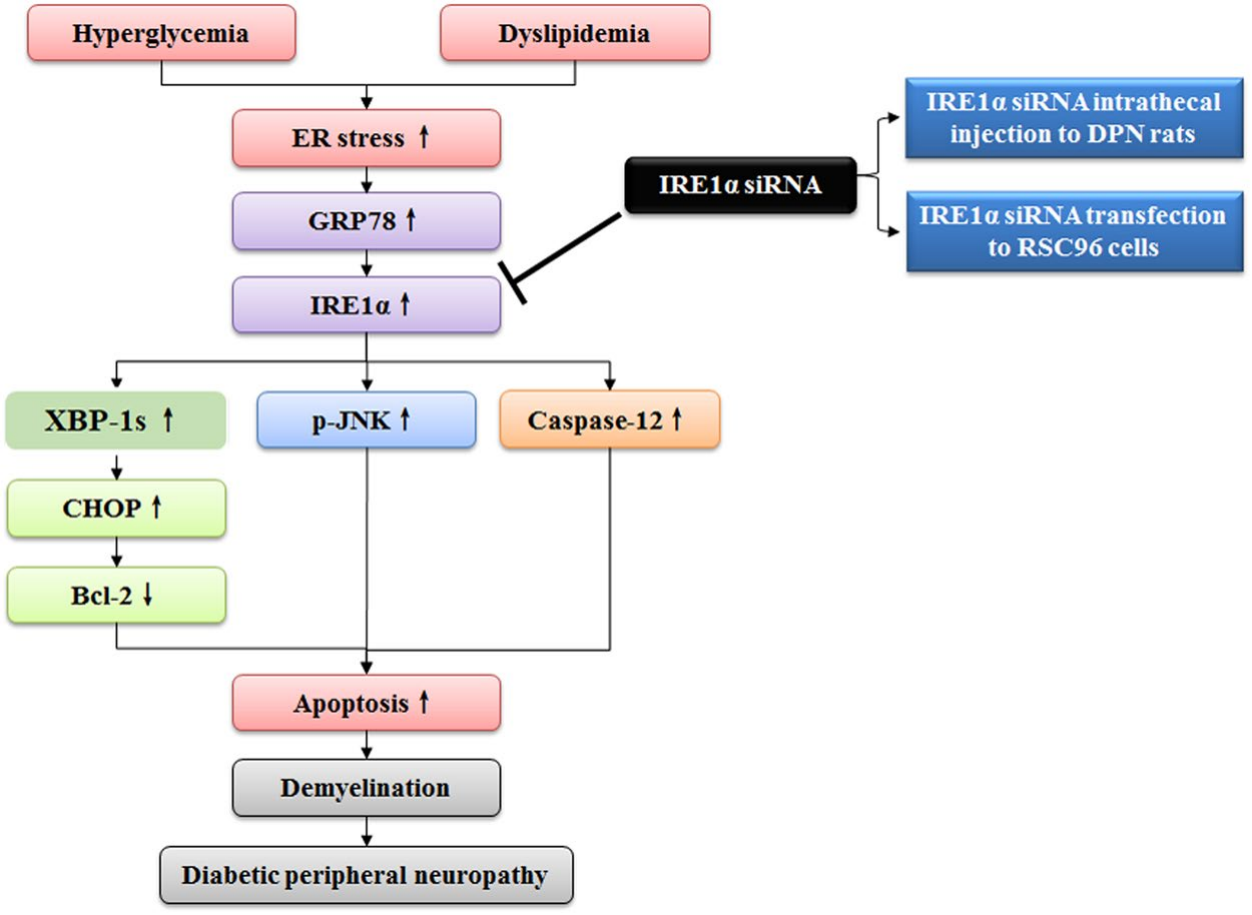
Schematic diagram depicting improvement of diabetic peripheral neuropathy due to relieved endoplasmic reticulum stress-induced apoptosis by IRE1α siRNA.

In this study, we investigated IRE1α related-apoptosis induced by ER stress in DPN both *in vivo* and *in vitro* to elucidate the effect of IRE1α siRNA on DPN. Our data highlight that IRE1α siRNA reduces the CHOP, Caspase-12 and JNK activation-related apoptosis, decreases the demyelination, thereby improving the DPN (Fig. 7), suggesting that IRE1α is a potentially valuable target for interventions aimed at improving DPN.

## Methods

### Animals and cells

Animal experiments procedures were approved by the Animal Care and Use Committee of the Capital Medical University (approval no. AEEI-2014-086). Male Sprague-Dawley (SD) rats weighing 180–220 g were obtained from the Experimental Animal Center at Capital Medical University, Beijing, China (SCXK 2012–0001) and were housed with a 12/12-hour light/dark cycle in a temperature and humidity-controlled environment. All experiments were performed in accordance with relevant guidelines and regulations.

RSC96 cells (CRL-2765) were purchased from the American Type Culture Collection (ATCC, cat no. CRL-2765) and were cultured in DMEM modified to contain 4 mM L-glutamine, 25 mM glucose, 1 mM sodium pyruvate, 1500 mg/L sodium bicarbonate, 10% FBS and 1% antibiotics at 37 °C in a humidified atmosphere of 5% CO_2_ and were passaged once every 3 days.

### Animal model of DPN and IRE1α siRNA intrathecal injection

Animals were divided into control, DPN, control plus IRE1α siRNA and DPN plus IRE1α siRNA groups. DPN was induced by feeding the animals with high-carbohydrate/high-fat diet and low-dose streptozotocin (STZ, S0130, Sigma). Specifically, rats in two DPN groups were fed with high-carbohydrate/high-fat diet (67% standard diet, 10% lard, 20% sucrose, 2.5% cholesterol and 0.5% cholate) for 4 weeks followed by treating with streptozotocin (35 mg/kg in 0.1 M citric acid buffer, pH 4.5, i.p.) The rats with fasting blood glucose level above 16.7 mM 1-week after STZ injection were considered diabetic and were continued feeding on the high-carbohydrate/high-fat diet for 12 weeks. Fasting blood glucose levels were measured every 2 weeks to monitor the persistence of diabetes and thermal perception thresholds (TPT) were measured every 4 weeks to track the occurrence of peripheral neuropathy.

At 12 weeks after induction of diabetes, intrathecal injection of IRE1 siRNA was carried out. Rats were anesthetized using chloral hydrate (i.p., 300 mg/kg). L4-L5 vertebrae were exposed and a sterile polyethylene tubing (PE-10 catheter) was implanted into the subarachnoid space according to the previously published^25^. Rats were allowed 24 hours to recover from surgery prior to treatment.

IRE1α siRNA (sc-270028, Santa Cruz, USA) was prepared immediately prior to administration by mixing the siRNA solution with a transfection regent (sc-29528, Santa Cruz, USA), in a ratio of 1: 4 (v/v). The final concentration of RNA as an RNA/lipid complex was 0.4 μg/μL. The IRE1α siRNA complex or transfection regent (10 μL) alone was injected in Control IRE1α siRNA transfected (Control + IRE1α siRNA), DPN IRE1α siRNA transfected (DPN + IRE1α siRNA) and Control, DPN groups using a 25 μL microsyringe, respectively. 10 μL saline solution was used to wash the catheter. Injections were given daily for 3 consecutive days^24,26^. Nerve conduction velocity test and blood, tissue harvest were carried out 24 hour after the last injection.

### Cells culture and transfection

RSC96 cells plated at a density of 2 × 10^5^cells/well in 6-well plate were allowed to adhere overnight, and then transfected with IRE1α siRNA according to the manufacturer’s instructions. Briefly, for each transfection, 100 pmols of siRNA and 6 µl transfection reagent were added to 100 µl siRNA transfection medium (sc-36868, Santa Cruz), gently mixed, incubated for 25 minutes at room temperature, then 1.0 ml siRNA transfection medium containing 200 µl siRNA transfection reagent mixture was added to the well. Cells were incubated 8 hours at 37 °C in a CO_2_ incubator, 1 ml normal growth medium containing 2 times the normal serum and antibiotics concentration (2× normal growth medium) was added without removing the transfection mixture. Cells were incubated for an additional 16 hours and the medium was aspirated and cultured in 25 mM or 150 mM glucose growth medium for 24 hours and 48 hours, respectively.

Cells were treated with 25 mM glucose, 150 mM glucose, 25 mM glucose IRE1α siRNA transfected (25 mM glucose + IRE1α siRNA) and 150 mM glucose IRE1α siRNA transfected (150 mM glucose + IRE1α siRNA). Cells in 25 mM glucose, 150 mM glucose groups were treated with siRNA transfection reagent alone (Supplementary Fig. 1).

### Measurement of nerve conduction velocity

Nerve conduction velocity was assessed using Functional Experiment System (BL-420s, Techman, China) as reported previously^27^. Before the measurement, rats were anesthetized with 10% chloral hydrate (i.p., 300 mg/kg). For measurement of MNCV, stimulation electrode was placed at the notch of sciatic nerve. Sciatic nerve was stimulated with single square wave pluses (1.2 V in intensity, 1 ms in width). For SNCV, recording site was located in the sciatic notch. Stimulation parameters were the same as MNCV methods.

### Morphometric analysis of sciatic nerve

After the conduction velocity test, the left sciatic nerve was isolated and cut into two segments. One segment (2 mm) was fixed in 2.5% glutaraldehyde at 4 °C, and sent to the Electron Microscopy Center of Institute of Capital Medical University for ultrastructure observation.

The other segment (1 cm) was fixed in 10% buffered formalin and processed for paraffin section. Sciatic nerve tissues were cut in slice of 5 μm thick. Sections were stained with Luxol fast blue, and microphotographs were captured using a light microscope (Nikon Eclipse 80i, Japan).

### Terminal dUTP nick-end labeling (TUNEL) assay

Apoptotic tissues were labeled using *in situ* cell death detection kit from Roche Company, according to manufacturer’s protocol. The nerve sections were dewaxed and followed by incubation with proteinase K for 15 min at room temperature. Following digestion, the end-labeling reaction was performed by adding TdT and dUT reaction mix onto the slides at 37 °C for 1 h at humidified chamber. After three PBS washes, converter-POD was added onto sections and incubated at 37 °C for 30 min at humidified chamber followed by reaction with DAB at room temperature for 10 min. Staining was observed using a light microscope.

### Immunofluorescence staining

The nerve sections were dewaxed, antigen repaired and blocked with 3% BSA. One hour later, sections were incubated in the following primary antibodies overnight at 4 °C: anti-IRE1α (sc-390960; 1: 50; Santa Cruz), anti-p-JNK (sc-6254; 1: 50; Santa Cruz) and rabbit anti-Caspase-12 (sc-5627; 1: 50; Santa Cruz). After rinsing, the sections were incubated with the fluorescein (FITC)-conjugated goat anti-mouse IgG (H+L) or FITC-conjugated goat anti-rabbit IgG (H+L) for 1 h at room temperature. Microphotographs were captured using the Nikon Eclipse 80i light microscope^7^.

### Western blotting

Sciatic nerves and RSC96 cells were lysed on ice in the RIPA buffer with protease inhibitor cocktail and phosphatase inhibitor cocktail for 30 min to extract total proteins. The proteins were analyzed with a bicinchoninic acid (BCA) protein assay kit (Biosynthesis, China). 30 μg/lane (sciatic nerve) and 20 μg/lane (RSC96 cell) were used for Western blot analysis as previously described^8,27^. The primary antibodies were as follows: mouse anti-IRE1α (sc-390960; 1: 1000; Santa Cruz), rabbit anti-P-IRE1α (ab48187; 1: 2000; abcam), mouse anti-XBP1 (sc-8015; 1: 1000; Abcam), mouse anti-GRP78 (sc-376768; 1: 1000; Santa Cruz), mouse anti-GADD153 (sc-7351; 1: 500; Santa Cruz), rabbit anti-Caspase-3 (ab13847; 1: 1000; abcam), rabbit anti-Caspase-12 (om273459; 1: 1000; Omnimabs), mouse anti-Bcl-2 (sc-7382; 1: 1000; Santa Cruz), mouse anti-Bax (sc-7480; 1: 1000; Santa Cruz), mouse anti-p-JNK (sc-6254; 1: 1000; Santa Cruz), and rabbit anti-PGP9.5 (ab108986; 1: 2000; Abcam). Mouse anti-β-actin (TA-09; 1: 20000; Zhongshan Goldenbridge) served as the internal control. The secondary antibodies were goat anti-mouse IgG-HRP (ZB-2305; Zhongshan Goldenbridge) and goat anti-rabbit IgG-HRP (ZB-2301; Zhongshan Goldenbridge). Western Chemiluminescent HRP Substrate and exposed to X-film to form image. The protein bands were quantitated with Image J software^27^.

### Apoptosis assay

An Annexin-FITC Apoptosis Detection Kit (AP101, Multisciences, China) was used to examine apoptosis according to the manufacturer’s instructions^8^. In brief, cells were added with 500 μL of binding buffer followed by staining with 5 μL of FITC-labeled annexin V and 10 μL of propidium iodide (PI) and incubated at room temperature for 5 min in the dark. BD LSRFortessa™ flow cytometer (BD Biosciences, San Jose, CA, USA) were used to analysis.

### Intracellular Ca^2+^ analysis

Fluo-3 AM (S1056, Beyotime, China) was used to examine intracellular Ca^2+^ levels according to the manufacturer’s instructions^28^. Cells were harvested and washed twice with PBS, then loaded with Fluo-3 AM (5 μM) for 30 min at 37 °C in the dark, and was analyzed using BD LSRFortessa™ flow cytometer.

### Statistical analysis

Data were expressed as the mean ± SEM. Statistical analysis was performed by unpaired Student’s t-test or One-way ANOVA followed by least significant difference (LSD) or Tamhane’s T2 analysis using SPSS 17.0. Values of *P* < 0.05 were statistically significant.

### Data availability

The datasets generated during and/or analyzed during the current study are available from the corresponding author on reasonable request.

## Electronic supplementary material

Supplementary information
