# Supplementary material for: IRE1α siRNA relieves endoplasmic reticulum stress-induced apoptosis and alleviates diabetic peripheral neuropathy in vivo and in vitro
Source: Sci Rep. 2018 Feb 7;8:2579. doi: 10.1038/s41598-018-20950-9 (PMC5803253; doi:10.1038/s41598-018-20950-9)
Supplement: Supplementary file 1 — Supplementary information [file 41598_2018_20950_MOESM1_ESM.pdf]

IRE1α siRNA relieves endoplasmic reticulum stress-induced apoptosis and alleviates diabetic peripheral neuropathy *in vivo* and *in vitro*

Weijie Yao[1,*], Xinwei Yang[1,*], Jiayue Zhu[1], Biane Gao[1], Haotian Shi[1], Liping Xu [1,#]

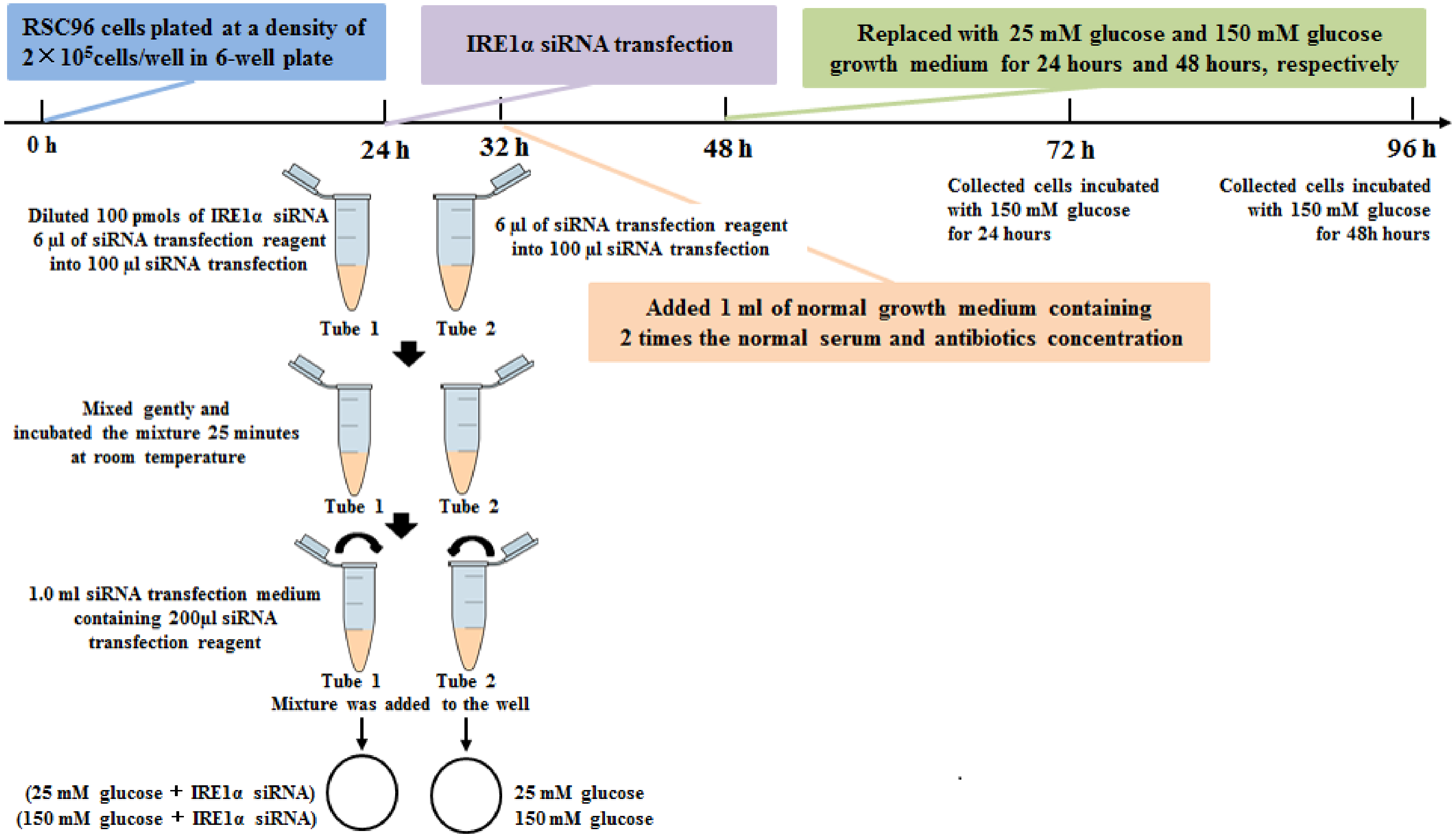

Supplementary Figure. 1 The procedure of RSC96 siRNA transfection
